# Toxinotyping, Antibiotic Resistance Profile, and In Vitro Bio-Control of *Clostridium perfringens* Type G Isolated from Chickens with Necrotic Enteritis by Lytic Bacteriophages

**DOI:** 10.3390/antibiotics15050453

**Published:** 2026-04-30

**Authors:** Hoang Minh Duc, Nguyen Thi Lan, Tran Thi Khanh Hoa, Cam Thi Thu Ha, Le Van Hung, Nguyen Van Thang, Hoang Minh Son

**Affiliations:** 1 Department of Veterinary Public Health, Faculty of Veterinary Medicine, Vietnam National University of Agriculture, Gia Lam, Hanoi 12400, Vietnam; 2 Laboratory of Veterinary Microbiology, Center of Research Excellence and Innovation, Vietnam National University of Agriculture, Gia Lam, Hanoi 12400, Vietnam; 3 Department of Pathology, Faculty of Veterinary Medicine, Vietnam National University of Agriculture, Trau Quy, Gia Lam, Hanoi 12400, Vietnam; 4 Key Laboratory for Veterinary Biotechnology, Vietnam National University of Agriculture, Gia Lam, Hanoi 12400, Vietnam; 5 Department of Anatomy and Histology, Faculty of Veterinary Medicine, Vietnam National University of Agriculture, Trau Quy, Gia Lam, Hanoi 12400, Vietnam

**Keywords:** *Clostridium perfringens*, necrotic enteritis, bacteriophages, bio-control, antibiotic resistance

## Abstract

**Background/Objectives:** Necrotic enteritis (NE), induced by *Clostridium perfringens*, is responsible for significant economic losses in the poultry industry worldwide. The growing restrictions on antibiotic use have driven the search for alternative strategies for disease control. The purpose of this study is to isolate and characterize lytic phages targeting multidrug-resistant *C. perfringens* type G recovered from chickens with NE. **Methods:** *C. perfringens* was isolated from chickens with NE using a culture method with selective TSC agar. Bacterial identification was carried out using biochemical tests and PCR. *C. perfringens* isolates were toxinotyped by PCR. Antibiotic susceptibility test was performed using the agar dilution method. Bacteriophages were isolated from chicken intestine samples collected from wet markets using the double-layer agar technique. Phage isolates were characterized by host range, one-step growth, stability, and whole genome sequencing. The efficacy of phage CPP8 in controlling multidrug-resistant *C. perfringens* type G was evaluated in GAM broth. **Results:** In this study, 16 *C. perfringens* strains were isolated from 100 chickens suspected of NE. Among these isolates, 10 (62.5%) belonged to type G, while the remaining 6 (37.5%) were type A. A total of 11 phages capable of lysing *C. perfringens* type G were isolated from the chicken intestine. Among them, phage CPP8 has the widest host range, short latent period, large burst size, and high stability. Moreover, the genome of CPP8 lacked genes related to antibiotic resistance, toxins, virulence factors, or lysogeny. Treatment with CPP8 resulted in a significant reduction in viable counts of *C. perfringens* at 37 °C. **Conclusions:** Our findings highlight phage CPP8 as a promising candidate for bio-control of multidrug-resistant *C. perfringens* type G.

## 1. Introduction

Necrotic enteritis (NE), caused by *Clostridium perfringens* (*C. perfringens*), has been recognized as one of the most important diseases, leading to significant economic losses in the poultry industry [1]. This disease is presented as an acute clinical or subclinical form [2]. The acute form of NE is characterized by typical symptoms, including depression, dehydration, ruffled feathers, diarrhea, and inappetence [3]. The mortality rate of this form increases for several days and can reach up to 50% [2,4]. The subclinical form of NE leads to chronic damage of the gut epithelial layer, thereby reducing nutrient absorption and growth performance [4]. In the United States, NE infection annually causes significant economic losses of 6 billion US dollars in the poultry industry due to high mortality, poor productivity, and treatment costs [5].

*C. perfringens* is an anaerobic, Gram-positive, spore-forming bacterium that is commonly found in the environment and in the digestive tract of humans and animals [6]. *C. perfringens* is classified into 7 toxin types (A–G) based on the production of 6 major lethal toxins: alpha (*α*), beta (*β*), epsilon (*ε*), iota (*ι*), enterotoxin (CPE), and NetB [7,8]. Historically, *C. perfringens* types A and C have been identified as the primary causes of NE, with type A known as the more virulent strain. However, the NetB toxin has been recently reported as the main NE-causing factor, resulting in the reclassification of NetB-producing *C. perfingens* type A into type G, a novel toxinotype capable of simultaneous production of *α*-toxin and NetB toxin [9,10].

Various antimicrobial agents have been administered daily in feed or water to control NE, leading to the development of antimicrobial resistance [11]. To address this challenge, prophylactic antimicrobial use has recently been restricted or banned in many countries, including the United States, Europe, and Vietnam [12,13]. As a result, the poultry industries in Canada and the USA have recently experienced an increase in the incidence of NE and other bacterial diseases [14,15].

Recently, bacteriophages have been increasingly considered a promising alternative to antimicrobials for controlling bacterial infection due to their suitable characteristics [16,17]. Phages are easily isolated as they are the most abundant biological entities on Earth [18,19]. They kill only harmful target bacteria without disturbing beneficial microorganisms in the intestinal tracts [19,20,21]. Phages are known as bacterial viruses, which do not have a mechanism to infect mammalian cells. Therefore, they are harmless to both humans and animals [22,23]. Unlike antibiotics, phages are self-replicating and self-limiting; several administrations are unnecessary [24]. This study aims to isolate and characterize phages capable of lysing multidrug-resistant *C. perfringens* type G recovered from chickens with NE in Vietnam.

## 2. Results

### 2.1. Isolation, Identification, and Toxinotyping of C. perfringens

**Figure 1:**
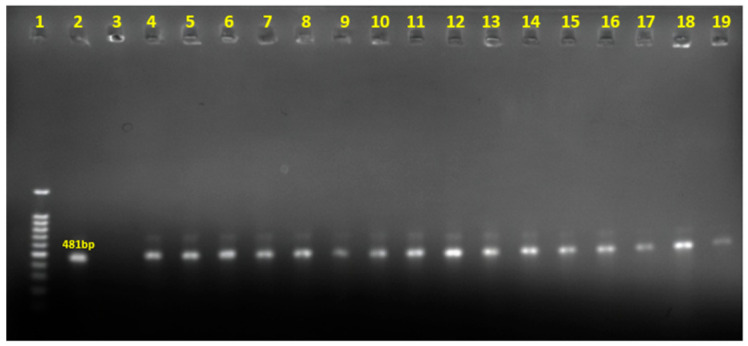
Detecting the *16S-rRNA* gene of *C. perfringens* by PCR. Lane 1: Marker; Lane 2: Positive control; Lane 3: Negative control; Lane 4–19: *C. perfringens* isolates.

A total of 16 chicken samples were positive for *C. perfringens* (Figure 1). To avoid duplicates, only one strain was isolated and preserved for each positive sample, corresponding to 16 *C. perfringens* strains.

**Figure 2:**
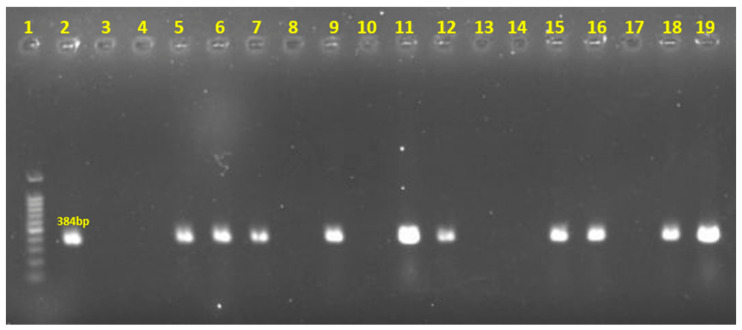
Detecting *netB* toxin genes of *C. perfringens* by PCR. Lane 1: Marker; Lane 2: Positive control; Lane 3: Negative control; Lane 4–19: *C. perfringens* isolates.

Among them, 10 (62.5%) isolates harbored both *cpa* and *netB*, while 6 (37.5%) only carried *cpa* (Figure 2). None of the isolates were positive for *cpb*, *cpb2*, *cpe*, *etx*, and *iap*. The results indicate that 10 (62.5%) out of 16 *C. perfringens* isolates were classified as type G, while 6 isolates belonged to type A.

### 2.2. Antibiotic Resistance of C. perfringens Isolates

The antibiotic resistance profile of *C. perfringens* isolates is shown in Table 1. The isolates showed a high rate of resistance to tetracycline (14/16; 87.5%), followed by clindamycin (9/16; 56.25%), ampicillin (7/16; 43.75%), chloramphenicol (7/16; 43.75%), and cefotaxime (6/16; 37.5%). By contrast, none of the isolates exhibited resistance to imipenem and cefoxitin. A total of 62.5% (10/16) of *C. perfringens* isolates were multidrug-resistant.

### 2.3. C. perfringens Phage Isolation

To isolate phages, a multidrug-resistant *C. perfringens* CP6 belonging to type G was exploited as a bacterial host. A total of 11 *C. perfringens* phages were isolated from 100 chicken intestine samples collected from wet markets in Hanoi, Vietnam. Of 11 isolated phages, 8 phages with clear plaques and high-titer stocks were selected for further characterization.

### 2.4. Characterization of Isolated C. perfringens Phages

#### 2.4.1. Host Range of Isolated Phages

Table 2 shows the lytic spectrum of 8 selected phages. Phage CPP8 had the widest lytic spectrum, lysing 12 (75%) of 16 *C. perfringens* strains tested. CPP3 and CPP7 also showed broad lytic spectrums, infecting 68.75% (11/16) and 62.5% (10/16) of *C. perfringens* isolates, respectively. On the contrary, phage CPP1 exhibited the narrowest host range, killing only 4 (25%) of 16 *C. perfringens* isolates. CP6 was used as a bacterial host for phage isolation; therefore, 100% (8/8) of the tested phages lysed this host. CP3 and CP16 were infected by 75% (6/8) of the phages examined, whereas CP7 was the most resistant host, being lysed only by CPP6. The broadest host range phage, CPP8, did not infect CP5, CP7, CP11, and CP14.

#### 2.4.2. Survival of the Phage CPP8 Under Various Conditions

**Figure 3:**
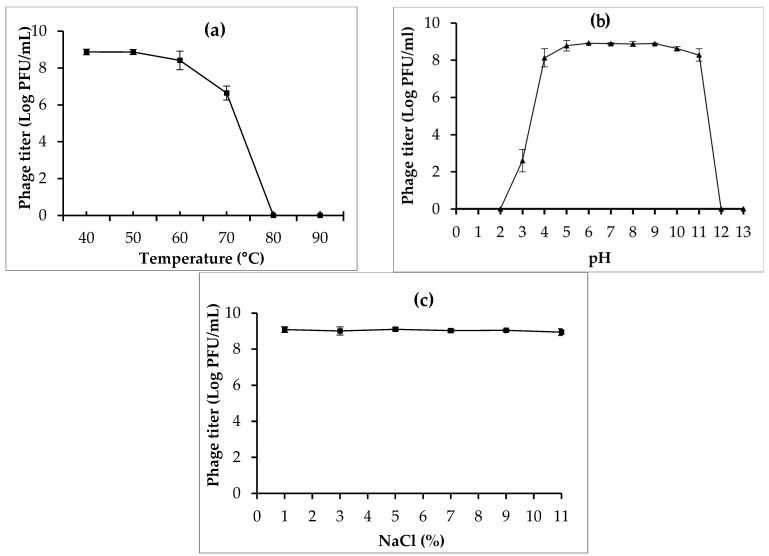
The viability of phage CPP8 under different temperature (**a**), pH (**b**), and NaCl (**c**) conditions. Error bars represent the standard deviation.

The ability of phage CPP8 to survive at different temperatures, pH, and NaCl conditions was shown in Figure 3. Overall, the results of the stability test indicated that phage CPP8 can survive over a wide range of temperatures (40–60 °C). However, phage viability was significantly reduced at 70 °C and completely inactivated at 80 °C. Similarly, phage CPP8 showed good stability across pH 4–11. At pH 3, CPP8 remained infectious, but phage titer decreased significantly. The complete inactivation of CPP8 occurred at pH 2 and pH 12. NaCl at concentrations of 1% to 11% showed no effect on the infectivity of CPP8 (Figure 3).

#### 2.4.3. One-Step Growth Curve of Phage CPP8

**Figure 4:**
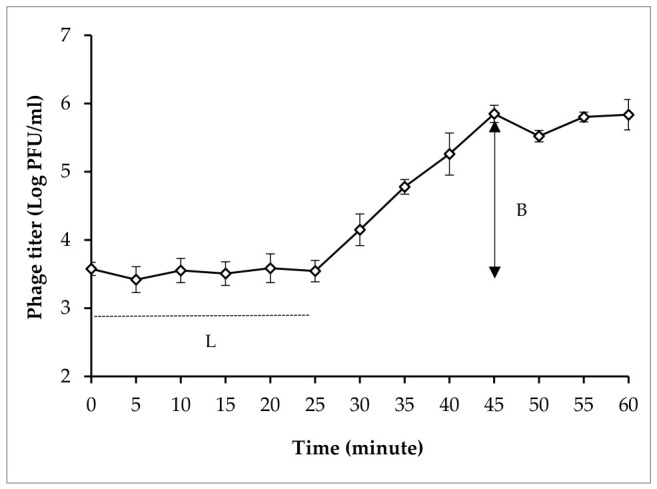
The replication curve of phage CPP8. L = latent period; B = burst size.

Figure 4 shows the replication curve of phage CPP8, with a short latent period of 25 min and a large burst size of 166 PFU/cell. These results suggest that phage CPP8 has the capability for rapid and prolific propagation.

#### 2.4.4. Genomic Analysis of Phage CPP8

**Figure 5:**
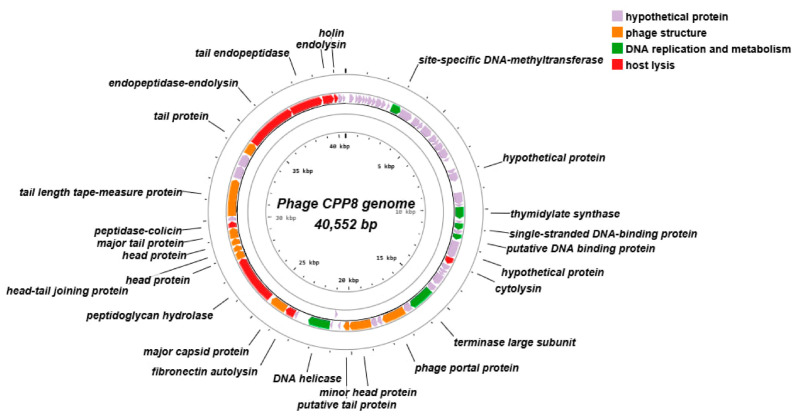
Genome map of phage CPP8.

**Figure 6:**
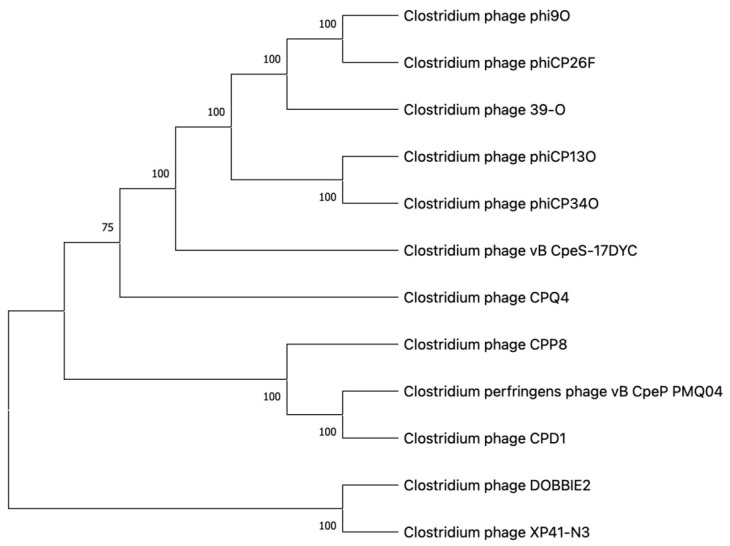
Phylogenetic tree generated using the whole genome sequence of 12 *Clostridium* phages, including *Clostridium* phage CPP8. The tree was generated in Mega 11.0 using the neighbor-joining method with *P*-distance values and a bootstrap replicate of 1000.

**Figure 7:**
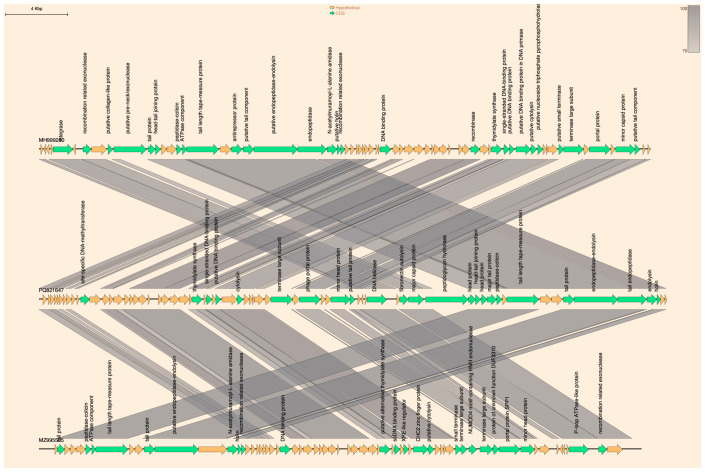
Genomic comparison of *Clostridium* phage CPP8 (middle), *Clostridium* phage CPD1 (top), and *Clostridium* phage vB_CpeP_PMQ04 (bottom). Whole genome alignment of the three phages visualized with GenoFig. Homologous regions were shown by grey shading. Percentages refer to the degree of amino acid identity between homologous genes.

The results of genomic sequence analysis indicate that phage CPP8 has a linear dsDNA genome consisting of 40,552 bp, with an overall G+C content of 30.6%. Genome annotation using the RAST server shows that the CPP8 genome encodes 68 ORFs but no tRNA genes. Analysis of ORF functions also reveals that 35.3% (24/68) of ORFs have putative functions, while 64.7% (44/68) were assigned hypothetical proteins. Three functional groups were identified, including: (i) phage structure (phage portal protein, minor head protein, major capsid protein, head-tail joining protein, head protein, major tail protein, terminase large subunit, putative tail protein, tail protein, and tail length tape-measure protein); (ii) DNA replication and metabolism (site-specific DNA-methyltransferase, thymidylate synthase, putative DNA-binding protein, and DNA helicase); (iii) host lysis (endolysin, endopeptidase–endolysin, tail endopeptidase, peptidoglycan hydrolase, holin, fibronectin autolysin, cytolysin, and peptidase–colicin). The genome of CPP8 did not carry any predicted antibiotic resistance, lysogeny, or virulence factor genes (Figure 5). According to BLASTN + 2.17.0 search, the genome of *Clostridium* phage CPP8 exhibited a high level of nucleotide sequence identity to those of *Clostridium* phage CPD1 (query cover 79%; identity 95.89%; accession number MH999280.1) and *Clostridium* phage vB_CpeP_PMQ04 (query cover 76%; identity 94.25%; accession number MZ995505.1). The result of phylogenetic analysis of phage CPP8 also indicated that this phage is a close relative of phage CPD1 and vB_CpeP_PMQ04 (Figure 6). Comparative analysis of whole-genome sequences (Figure 7) demonstrated that CPP8 is relatively similar to phages CPD1 and vB_CpeP_PMQ04. The distinct regions in the CPP8 genome compared to these two phages were ORFs encoding site-specific DNA-methyltransferase, terminase large subunit, peptidoglycan hydrolase, and several hypothetical proteins. The main difference between the genomes of CPP8 and CPD1 is that genes associated with lysogeny (integrase), as seen in the CPD1 genome, were not found in the CPP8 genome. Figure 7 also indicated that CPP8 carried more genes related to host lysis (endolysin, endopeptidase–endolysin, tail endopeptidase, peptidoglycan hydrolase, holin, fibronectin autolysin, cytolysin, and peptidase–colicin) than CPD1 and vB_CpeP_PMQ04, suggesting that CPP8 may have stronger lytic activity.

### 2.5. The Effect of Phage CPP8 on the Growth of C. perfringens

**Figure 8:**
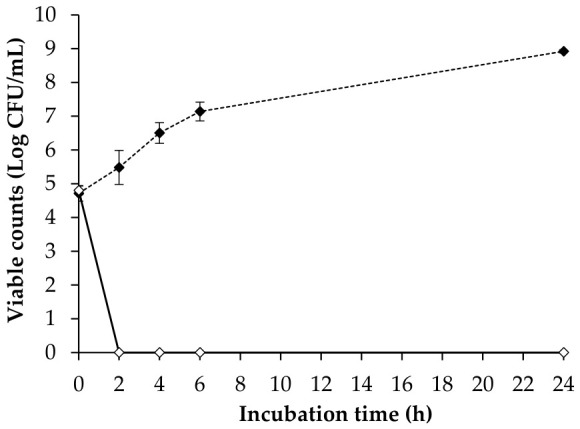
The lytic activity of phage CPP8 against *C. perfringens* in GAM broth. *C. perfingens* CP6 was cultured in 5 mL of GAM broth at 10^5^ CFU/mL without phage CPP8 (dashed line) and with phage CPP8 at 10^8^ PFU/mL (solid line). Error bars represent standard deviations.

The inactivation of *C. perfringens* CP6 in GAM broth at 37 °C by phage CPP8 is represented in Figure 8. Overall, the application of phage CPP8 resulted in significant decreases in viable counts of CP6 compared to untreated control at 2, 4, 6, and 24 h (*p* < 0.5). Without the addition of phage CPP8, the viable counts of CP6 increased gradually and reached a stationary phase at 24 h. In the phage treatment group, viable counts of CP6 were significantly declined to below the detection limit (< 10 CFU/mL) after 2 h of incubation (*p* < 0.5). At the end of the experiment (at 24 h), CP6 regrowth did not occur in the phage-treated group.

## 3. Discussion

The development of NE in chickens is attributed to the production of toxins by *C. perfringens.* The bacteria can produce approximately 20 different toxins associated with NE development [25]. The identification of NE-causing toxins and their type is vital for the development of NE vaccines. Type G, producing both *α*-toxin and NetB toxins, was known as the main type causing NE [10]. The role of α-toxin in the development of necrotic enteritis (NE) in chickens remains controversial. It was reported that a mutant *C. perfringens* strain lacking *α*-toxin retained its virulence and was still capable of causing NE in an experimental chicken model [26]. Similarly, another study found that the severity of intestinal lesions in chickens induced by *α*-toxin-deficient mutants was comparable to that of the wild-type strain. Refs. [9,26,27] found that a novel toxin, NetB, is an important virulence factor in the pathogenesis of necrotic enteritis in chickens. The toxin protein shared a low amino acid sequence identity with the *β*-toxin of *C. perfringens*, which is responsible for mucosal necrosis of the small intestine in humans and animals. NetB null mutants have not been shown to cause NE in chickens. By contrast, chickens challenged with the wild-type complemented with a wild-type netB gene developed NE [9,27]. A variety of studies screening for the *netB* gene in *C. perfringens* strains from many countries have shown a strong correlation between the presence of the *netB* gene and isolates from diseased chickens [28]. A study by Keyburn et al. (2010) found that *netB* was present in 70% (31/44) of strains recovered from NE-affected chickens in Belgium, Denmark, Australia, and Canada [29]. Also, *netB*-positive strains accounted for 77% of chicken necrotic enteritis isolates in Australia [9]. Similar findings were found in a study conducted in the United States, 82.1% (119 of 145) of isolates from dead chickens with NE carried *netB* [30]. A high detection rate (91%; 31/34) of *netB* was also observed in *C. perfringens* strains isolated from NE-specific organ lesions of chickens in Sweden [31]. Another study in Canada reported that 95% (39/41) of isolates from broilers with NE were *netB*-positive [32]. In our study, 10 (62.5%) of 16 *C. perfringens* strains isolated from NE-afflicted chickens harbored *netB* and belonged to type G. The incidence of *netB* in this study was lower than that observed in previous studies mentioned above, but higher than in other studies conducted in Vietnam, Italy, Korea, and Iran [28]. Thi et al. (2021) [33] reported that the presence rate of *netB* in *C. perfringens* strains isolated from intestinal samples of diseased chickens in Vietnam was 45% (18/40). A study in Italy showed that *netB* was detected in 16 (53.3%) out of 30 *C. perfringens* strains recovered from chickens affected by NE [34]. In Korea, the rate of *netB* in *C. perfringens* isolates from diseased chickens was 47.1% (8/17) [35]. In Iran, 8 (17.8%) of 45 *C. perfringens* strains isolated from chickens clinically suspected of NE were positive for *netB* [36]. In the present study, 6 (37.5%) of 16 *C. perfringens* isolates were *netB*-negative and belonged to type A. This is in agreement with previous studies mentioned above, which indicated that not all (100%) *C. perfringens* isolated from chickens with NE harboured *netB.* It cannot be ruled out that *netB*-negative *C. perfringens*, a part of the commensal, anaerobic flora in the intestinal tract of chickens, may affect the isolation process [37]. The other possibility is that *C. perfringens* type A, in combination with other factors, may induce clinical signs and pathological lesions resembling those of necrotic enteritis [38]. Earlier studies reported that birds challenged with a *netB*-negative *C. perfringens* type A developed typical NE gross lesions when temporary starvation was applied as a predisposing factor [38].

The widespread use of antimicrobials for growth promotion, prevention, and treatment of poultry diseases has been attributed to the rapid development of antimicrobial resistance [39]. It has been reported that the production of 1 kg of live chicken in Vietnam uses approximately 77.4 mg of in-feed antimicrobials [40]. In this study, high rates of resistance were observed against tetracycline, clindamycin, chloramphenicol, and ampicillin. This is likely due to the intensive use of these antimicrobials in chicken production in Vietnam, resulting in selective pressure for resistance [40,41]. In addition, bacteria may develop resistance to antimicrobial agents even without prior exposure, as antibiotic resistance genes located in mobile genetic elements, including plasmids, transposons, and integrons, can horizontally transfer between bacterial populations [42].

For almost 100 years, antibiotics have been the most powerful weapons against bacterial infections in humans and animals; however, the rapid emergence of antibiotic-resistant bacteria has emphasized the urgent need for alternatives [16,43]. Several alternatives, including probiotics, phytogenics, enzymes, organic acids, and phages, have been proposed to replace antibiotics in livestock [44,45]. Among these approaches, phages have gained considerable attention as a promising alternative to antibiotics for maintaining animal health and ensuring productivity due to their multiple advantages. Phages can directly kill pathogenic bacteria through the lytic infection cycle, in which the virus replicates within the bacterial cell and ultimately leads to cell lysis, releasing progeny phages that continue infecting neighboring hosts [46,47,48], while probiotics primarily exert their antibacterial activity by competing with pathogenic bacteria for adhesion sites and nutrients, as well as inhibiting their growth through the production of antimicrobial compounds [49]. Phages differ from phytogenics, enzymes, and organic acids in that they eliminate only harmful bacteria without a negative impact on microbiota [50,51]. In addition, phages can destroy biofilms and kill multidrug-resistant bacteria [52,53,54]. Furthermore, the relatively simple isolation, propagation, and large-scale production of bacteriophages make them a cost-effective alternative to conventional antibiotics [55].

Although the potential of phages in controlling bacterial infection in poultry has been documented in several studies, their efficacy remains inconsistent across studies and may depend on factors such as administration route, dosage, selected phages, and bacterial strain [44,56,57,58,59]. The isolation and selection of suitable phages are key steps to ensure the success of phage therapy [60]. A panel of bacterial hosts is crucial for isolating and determining the host range of desired phages, as pathogenic strains circulating in the field may not be susceptible to phages isolated using laboratory reference strains as hosts. Pathogenic strains isolated from different geographical regions are likely to have distinct genetic characteristics, virulence factors, and phage receptors, which can influence phage–host interactions. Therefore, locally isolated pathogenic strains should be used as hosts for phage isolation and characterization to obtain phages with strong lytic activity and wide-host ranges [18,61]. In our study, 16 *C. perfringens* strains were isolated from chickens with NE. Multidrug-resistant *C. perfringens* type G, CP6, isolated from a chicken with severe NE lesion, was used as a host for phage isolation. *C. perfringens* phage, CPP8, exhibited the broadest host range against 16 *C. perfringens* isolates, which were selected for further characterization. In addition to lytic activity and host range, stability under varying environmental conditions was an important criterion for phage selection, as environmental factors such as temperature, pH, and NaCl can significantly affect phage viability, infectivity, and practical use in livestock [62]. In the present study, the stability of phage CPP8 was investigated under various physicochemical conditions. Phage CPP8 remained highly stable at temperatures between 40 and 60 °C, indicating its ability to tolerate the physiological temperature of poultry. This thermal resistance also suggests that CPP8-based formulations may maintain viability during storage at room temperature, which is beneficial for the commercialization and practical application of CPP8. Similarly, this phage showed great infectivity at pH ranges of 4 to 11, which is consistent with the previous studies, reporting that the pH ranges of *C. perfringens* phages were usually from 4 to 11 [63,64]. The findings in the present study indicate that phage CPP8 can pass through the gizzard (pH 2.33~3.52) and reach the intestines (pH 6.25~7.21) of chickens to exert antibacterial activity against target pathogens [63]. In addition, microencapsulation has been proposed as a novel approach to protect phage particles from gastrointestinal conditions and improve their viability in the intestinal tract of chickens [65,66]. In this study, the intestine of healthy chickens was selected as a source for phage isolation, with the expectation that isolated phages may be well adapted to the gastrointestinal environment of poultry, particularly the acidic conditions in the gizzard. Similar to the previous studies, the present study also proved that phages specific to foodborne pathogens were abundant in chicken meat and internal organs [52,67,68,69]. Phage CPP8 also exhibited resistance to high NaCl concentrations up to 11%, indicating high stability under osmotic stress and highlighting its potential for practical use in livestock. In addition to high environmental tolerance, other biological characteristics, such as a short latent period and a large burst size, should be considered during phage selection. The results of phage characterization showed the latent period and burst size of phage CPP8 were 25 min and 166 PFU/cell, respectively. This short latent period of 25 min enables phage CPP8 to infect and lyse host bacterial cells rapidly. While a large burst size of 166 PFU/cell enables the production of numerous phage progeny from each infected cell, thereby accelerating the phage population increase at the site of infection.

Genetic safety is considered a key requirement for phage application. The results of whole genome sequencing demonstrate that the genome of phage CPP8 did not contain any genes associated with toxins, virulence factors, antibiotic resistance, or lysogeny, indicating that this phage is safe for practical use. Genomic analysis of phage CPP8 also reveals that the phage harbored multiple genes responsible for host lysis (endolysin, endopeptidase–endolysin, tail endopeptidase, peptidoglycan hydrolase, holin, fibronectin autolysin, cytolysin, and peptidase–colicin), suggesting that phage CPP8 may have strong lytic activity. These findings align with the *in vitro* test, which showed that phage CPP8 reduced the viable counts of *C. perfringens* CP6 to below the detection limit, and bacterial regrowth did not occur even after 24 h of incubation at 37 °C. The bacterial regrowth was attributed to the emergence of phage resistance, which hinders the wide use of phages [48]. Combining multiple phages to produce a cocktail has been proposed as a simple approach to reduce the risk of the emergence of phage-resistant bacteria [70]. However, the use of a phage cocktail does not always guarantee greater efficacy as the competition among the phages for the same receptors on the surface of host cells may occur [71,72]. Several studies have previously reported the failures of phage cocktails in preventing bacterial regrowth or the worse effectiveness of multiple phage applications in comparison with a single one [73,74,75].

## 4. Materials and Methods

### 4.1. Isolation and Identification of C. perfringens from Chicken with Necrotic Enteritis

Chickens suspected of NE were marked for monitoring the clinical signs (reduced appetite, ruffled feathers, dark colored diarrhea, depression) at farms in Vietnam. Upon death, the chickens were immediately transported to the laboratory for analysis. In the laboratory, a total of 100 chickens collected from different chicken flocks were necropsied to obtain an intestinal sample with typical lesions, including a thin intestinal wall, gas distension, confluent small-intestinal mucosal necrosis, and depressed ulcers on the mucosal surface. A portion of the intestinal sample (1 g) was then cultured in 5 mL of Fluid Thioglycollate Medium (TGM, Oxoid Ltd., Basingstoke, UK) and incubated at 42 °C in an anaerobic jar (Oxoid Ltd., UK). Following the incubation, the sample was serially diluted with buffered phosphate saline (BPS). An appropriate dilution (1 mL) was pipetted into a Petri dish. Molten soft tryptose sulfite cycloserine agar (TSC; Oxoid Ltd., UK) containing 5% egg yolk and perfringens selective supplement was then poured onto the Petri dish, left for 3–5 min at 24 °C to solidify, before a second layer of TSC agar was used to cover the first layer. After incubating anaerobically for 24 h at 37 °C, well-separated, typical *C. perfringens* colonies with black colour and cloudy halos were chosen for biochemical test using the API 20A kit (Biomerieux, Marcy-l’Étoile, France). Further confirmation was performed by using PCR to detect the species-specific *16S-rRNA* gene following the method described previously by Tonooka et al. (2005) [76]. PCR-positive *C. perfringens* strains were preserved at −86 °C.

### 4.2. Toxinotyping of C. perfringens Isolates

The isolated *C. perfringens* strains were subjected to PCR to detect toxin genes (*cpa*, *cpb*, *etx*, *iap*, *cpe*, *netB*) according to the previously described method [9,77]. DNA extraction from *C. perfringens* isolates was performed using the GeneJet Genomic DNA Purification Kit (Thermo Fisher Scientific, Vilnius, Lithuania) following the guidelines of the manufacturer.

Multiplex PCR reaction for the detection of *cpa*, *cpb*, *etx*, *iap*, and *cpe* was carried out in 25 µL of mixture consisting of 2.5 μL of 10× PCR Buffer, 10 µL of 1 mM dNTPs, 5 µL of 1U Taq polymerase, 0.25 µL of 25 μM of each primer, 2 μL of DNA template, and 2.5 µL of deionized water. PCR amplification of the toxin genes was performed in the thermal cycling machine (Biorad T100, BioRad Laboratories, Hercules, CA, USA) with initial denaturation at 94 °C for 2 min, followed by 34 cycles at 94 °C for 1 min, 55 °C for 1 min, and 72 °C for 1 min, and final extension at 72 °C for 10 min. After separation on a 2% agarose gel, the PCR product was visualized under ultraviolet light using a Bio-Rad Molecular Imager GelDoc XR (Bio-Rad Laboratories, Hercules, CA, USA).

The detection of *netB* gene was performed by PCR reaction in a 25 µL mixture composed of 2.5 μL of 10× PCR Buffer, 5 µL of 1 mM dNTPs, 5 µL of 1U Taq polymerase, 1 µL of 5 μM of each primer, 2 μL of DNA template, and 8.5 µL of deionized water. The PCR program for the *netB* amplification included an initial denaturation at 94 °C for 2 min, followed by 35 cycles of denaturation at 94 °C for 30 s, annealing at 55 °C for 30 s, extension at 72 °C for 1 min, and a final extension at 72 °C for 12 min. The analysis of the PCR product was subsequently carried out using the same method as described earlier.

### 4.3. Antibiotic Susceptibility of C. perfringens Isolates

The antibiotic resistance of *C. perfringens* isolates was tested by the agar dilution method, according to the Clinical and Laboratory Standards Institute (CLSI) guidelines [78]. The lowest concentration of an antimicrobial that prevents the visible growth of the isolates is considered the minimum inhibitory concentration (MIC). A total of 7 antibiotics, including ampicillin, cefoxitin, cefotaxime, imipenem, tetracycline, chloramphenicol, and clindamycin, were selected to determine the antibiotic resistance of the isolates, with *Clostridium difficile* ATCC 700057 as the quality control strain. Multidrug-resistant (MDR) strains were the isolates showing resistance to at least one agent in three or more antimicrobial classes.

### 4.4. Isolation, Purification, and Propagation of C. perfringens Phages

A total of 100 chicken intestinal samples were purchased from wet markets in Gia Lam, Hanoi, for isolating phages using the double-layer agar technique as described by Duc et al. (2018) and a *C. perfringens* CP6 as bacterial host [67]. Each chicken intestinal sample (25 g) was aseptically cut into small pieces and homogenized with 100 mL of Gifu Anaerobic Broth (GAM; Shimadzu Diagnostics Corporation, Tokyo, Japan) supplemented with 10 mM CaCl_2_ and bacterial host, incubated anaerobically at 37 °C for 18–24 h. After incubation, the enrichment broth (10 mL) will be centrifuged at 12,000 rpm for 10 min and passed through a 0.45 µm pore-size sterile membrane filter (Merck Millipore, Carrigtwohill, Ireland) to induce phage lysate. The lysate was serially diluted with saline magnesium (SM) buffer (0.05 M Tris-HCl buffer, containing 0.1 M NaCl, 0.008 M MgSO_4_, and 0.01% gelatin, pH 7.5), mixed with the bacterial culture, molten agar, and poured onto the surface of Brain Heart Infusion (BHI; Becton, Dickinson and Company, Franklin Lakes, NJ, USA) agar plates. These double-layer agar plates were incubated at 37 °C for 24 h. After incubation, large, round, clear plaques were picked by micropipette tip, diluted in SM buffer, mixed with the target bacterial culture and molten agar, and poured onto BHI agar plates. The plate was then incubated anaerobically at 37 °C for 24 h. Following the incubation, the single plaque was collected for another round of plaque purification. At least 3 rounds of purification were conducted to produce a pure phage. After purification, phage isolates were propagated to obtain phage stocks using the method previously described by Duc et al. (2018) [67]. Phage stocks were then kept at 4 °C for phage characterization.

### 4.5. Characterization of C. perfringens Phages

#### 4.5.1. Lytic Spectrum of *C. perfringens* Phages

The lytic spectrum of *C. perfringens* phages was examined on 16 *C. perfringens* isolates using the spot test assay previously described by [67,79]. The molten top agar was inoculated with the bacterial host before being poured on the bottom agar. For solidification, the double-layer agar was left at room temperature for 5 min. Afterwards, the phage suspension (10 µL) was spotted onto the surface of the double-layer agar plate. After incubation at 37 °C for 24 h, the presence of plaques on the agar plates was observed.

#### 4.5.2. The Replication Curve of *C. perfringens* Phage CPP8

The replication of *C. perfringens* phage CPP8 using CP6 as host was determined according to the previously described method [67]. One milliliter of the phage suspension (10^6^ PFU/mL) was used to infect 1 mL of bacterial culture (CP6; 10^8^ CFU/mL) to achieve the multiplicity of infection (MOI) of 0.01. The mixture was then incubated for 10 min at 37 °C before centrifuging at 10,000× *g* for 30 s at room temperature. After centrifugation, the pellet was collected and resuspended in 10 mL of GAM broth. The suspension was then kept in a water bath at 37 °C with shaking for 60 min. A sample (100 μL) was withdrawn every 5 min to determine phage titer using the double-layer agar technique. The latent period and burst size of phage CPP8 were calculated using the previously described formula [80].

#### 4.5.3. The Viability of the Phage CPP8 Under Various Conditions

The stability of phage CPP8 was tested following the previously described method [52]. To investigate the heat tolerance of phage CPP8, 100 μL of phage suspension at a concentration of 5 × 10^10^ PFU/mL was transferred into 5 mL of preheated SM and incubated in a shaking water bath for 30 min at a temperature range of 40 °C to 90 °C. The viability of phage CPP8 was then determined using the double-layer agar technique as described above. Similarly, phage CPP8 was added into 5 mL of SM previously adjusted to various pH values (2–13) to examine its survival for 60 min at 37 °C. The infectivity of CPP8 in NaCl at different concentrations (1–11%) was also tested at 37 °C for 60 min.

#### 4.5.4. Genomic Characterization of Phage CPP8

In this study, the Phage DNA Isolation Kit (Norgen Biotek, Thorold, ON, Canada) was used for the DNA extraction of phage CPP8. The whole genome of phage CPP8 was sequenced using Nanopore technology and assembled using CLC Genomics Workbench v. 24.0.2. The annotation of open reading frames (ORFs) of the assembled genome was conducted using the RAST server [81] and BLASTP [82]. The tRNAscan-SE was then used to scan the putative tRNAs encoding genes of the phage CPP8 genome [83] and ARAGORN [84]. The genes associated with antimicrobial resistance and virulence factors were screened using the ResFinder-3.1 [85] and VirulenceFinder-2.0 [86]. The fully sequenced genome of phage CPP8 has been deposited in the GenBank database under accession number PQ821647. The phylogenetic tree was created by Mega 11.0 using the neighbor-joining method with *P*-distance values and a bootstrap replicate of 1000 [87]. Finally, Proksee was applied to generate the genome map of phage CPP8 [88].

#### 4.5.5. The Effect of Phage CPP8 on the Viability of *C. perfringens*

The efficacy of phage CPP8 in reducing viable counts of *C. perfringens* CP6 was determined following the previously described method [89]. Briefly, the bacterial culture of CP6 (100 µL; 5 × 10^6^ CFU/mL) was inoculated into 5 mL of GAM broth to obtain a final concentration of approximately 10^5^ CFU/mL. For the treatment group, the bacterial suspension was then treated with 100 μL of phage suspension (5 × 10^9^ PFU/mL) and incubated at 37 °C. SM (100 µL) was used instead of the phage suspension in the control group. After incubation for 2, 4, 6, and 24 h, 100 μL of the sample was withdrawn to determine the viability of CP6. Briefly, 100 µL of the sample was serially diluted with PBS. A portion of proper dilutions (100 µL) was poured into a Petri dish and then covered with molten soft TSC. When the agar plate was solidified, a second layer of TSC agar was poured onto the agar plate and incubated anaerobically at 37 °C for 48 h. Following the incubation, the viable counts of CP6 were enumerated.

### 4.6. Statistical Analysis

Each experiment was replicated at least 3 times. The data were shown as mean values and standard deviation of the mean (SD). Statistically significant differences between the treatment groups and control groups were determined using Student’s *t*-test. Differences were considered statistically significant at *p* < 0.05.

## 5. Conclusions

In conclusion, 16 *C. perfringens* strains were isolated from the intestines of chickens with NE, with a high proportion belonging to type G. Additionally, 11 phages specific to *C. perfringens* were successfully recovered from the chicken intestine samples collected from wet markets. Among these, phage CPP8 exhibited the broadest lytic spectrum, a short latent period, a large burst size, and high environmental stability. Genomic analysis confirmed that CPP8 carried no genes associated with antibiotic resistance, toxins, lysogeny, or virulence. Moreover, phage application significantly reduced the viable counts of *C. perfringens*. Overall, these results demonstrated the potential of phage CPP8 as an effective bio-control agent against multidrug-resistant *C. perfringens* type G.

## Figures and Tables

**Table 1:** Antibiotic resistance profile of *C. perfringens* isolated from chickens with NE.

Antibiotic Class		Antibiotic Agent	Detection Range	MIC_50_ (µg/mL)	MIC_90_ (µg/mL)	No. of Resistant Isolates	Resistant Rate (%)
Beta-lactams	Penicillins	ampicillin	0.5–8	1	8	7	43.75
	Cephalosporins	cefoxitin	0.125–16	2	16	0	0
		cefotaxime	0.5–128	16	64	6	37.5
	Carbapenems	imepenem	0.125–8	0.5	4	0	0
Tetracyclines		tetracycline	2–64	32	32	14	87.5
Phenicols		chloramphenicol	1–64	16	64	7	43.75
Lincosamides		clindamycin	0.25–32	8	16	9	56.25

**Table 2:** Lytic spectrum of isolated phages.

Bacterial Isolate ID	Toxin Genes	Toxinotype	Isolated Phages								No. of Susceptible Phages
			CPP1	CPP2	CPP3	CPP4	CPP5	CPP6	CPP7	CPP8	
CP1	*cpa*	A	+	−	−	−	−	−	−	+	2
CP2	*cpa*, *netB*	G	−	−	+	+	+	−	+	+	5
CP3	*cpa*, *netB*	G	+	+	+	−	+	−	+	+	6
CP4	*cpa*, *netB*	G	−	−	+	+	+	−	+	+	5
CP5	*cpa*	A	−	+	−	+	−	−	−	−	2
CP6	*cpa*, *netB*	G	+	+	+	+	+	+	+	+	8
CP7	*cpa*	A	−	−	−	−	−	+	−	−	1
CP8	*cpa*, *netB*	G	−	−	+	−	−	+	+	+	4
CP9	*cpa*, *netB*	G	−	−	+	+	+	−	+	+	5
CP10	*cpa*	A	−	−	+	−	+	−	+	+	4
CP11	*cpa*	A	−	+	−	+	−	−	−	−	2
CP12	*cpa*, *netB*	G	−	−	−	+	+	−	+	+	4
CP13	*cpa*, *netB*	G	−	+	+	−	+	+	−	+	5
CP14	*cpa*	A	−	+	+	−	−	−	−	−	2
CP15	*cpa*, *netB*	G	−	+	+	−	−	+	+	+	5
CP16	*cpa*, *netB*	G	+	+	+	−	−	+	+	+	6
Total of infected strains			4	8	11	7	8	6	10	12	
Infected rate (%)			25.00	50.00	68.75	43.75	50.00	37.50	62.50	75.00	

(+): lysis; (−): no lysis.

## Data Availability

The data that support the findings of this study are available from the corresponding author upon reasonable request.
